# Late-Onset Manic Episode: Bipolar Disorder or Organic Secondary Mania ? A Case Report

**DOI:** 10.1192/j.eurpsy.2026.11200

**Published:** 2026-07-31

**Authors:** G. Ltaief, M. Ben Mbarek, I. Betbout, A. Karmous, F. Zaafrane, A. Mhalla

**Affiliations:** Department of Psychiatry, Fattouma Bourguiba University Hospital, Monastir, Tunisia

## Abstract

**Introduction:**

Mania has traditionally been considered a primary mood disorder, most often occurring in the context of either bipolar disorder or, less commonly, unipolar mania. More recent evidence demonstrates the occurrence of episodes of secondary mania attributable to identified organic, metabolic, pharmacologic, or neurologic processes. Within neurological causes, focal brain lesions are especially prevalent in the production of manic symptoms.

**Objectives:**

To demonstrate, using a clinical case and in the context of current literature, the difficulties encountered in differentiating between primary mania and secondary mood disorders in late-onset mania.

**Methods:**

Clinical case report and literature review.

**Results:**

Mr. A., a 48-year-old man with no relevant past psychiatric history or family psychiatric history, presented with his first-ever manic episode, demonstrating an elevated mood, grandiose and persecutory delusions, disinhibition (including coprolalia), and excessive spending on unrealistic business venture ideas. A psychiatric evaluation confirmed classical manic features. Due to the relatively late age of onset, he was subsequently evaluated for an organic cause to the episode. The initial CT scan was negative. The brain MRI revealed a nodular FLAIR hyperintense lesion in the periventricular white matter in the right medial frontal region with gadolinium enhancement; however, lumbar puncture was essentially normal (no oligoclonal bands noted). Autoimmune screens (e.g., anti-AQP4 and anti-MOG antibodies) were negative, examination of his systemic systems revealed no evidence of autoimmune or infectious disease, including neurological examination. Although multiple sclerosis was initially suspected, this diagnosis was excluded as there was no dissemination in time or space, CSF was normal, and the immunology was negative. However, we could not completely exclude secondary mania, stemming from an underlying organic cause.

The patient was admitted to the hospital, and his treatment regime included olanzapine. He made a good clinical progress as his mood was gradually stabilized, and delusional symptoms disappeared after three weeks of treatment. His treatment was subsequently optimized, reducing olanzapine gradually and switching to valproate sodium 1 g/day with close psychiatric monitoring.

Late-onset bipolar disorder is uncommon and generally requires exclusion of secondary diagnoses, such as multiple sclerosis, CNS infections, autoimmune encephalitis or frontotemporal dementia. Lesions leading to demyelination in the frontal lobe may disrupt fronto-limbic circuits potentially explaining the mood symptoms.

**Image 1:**
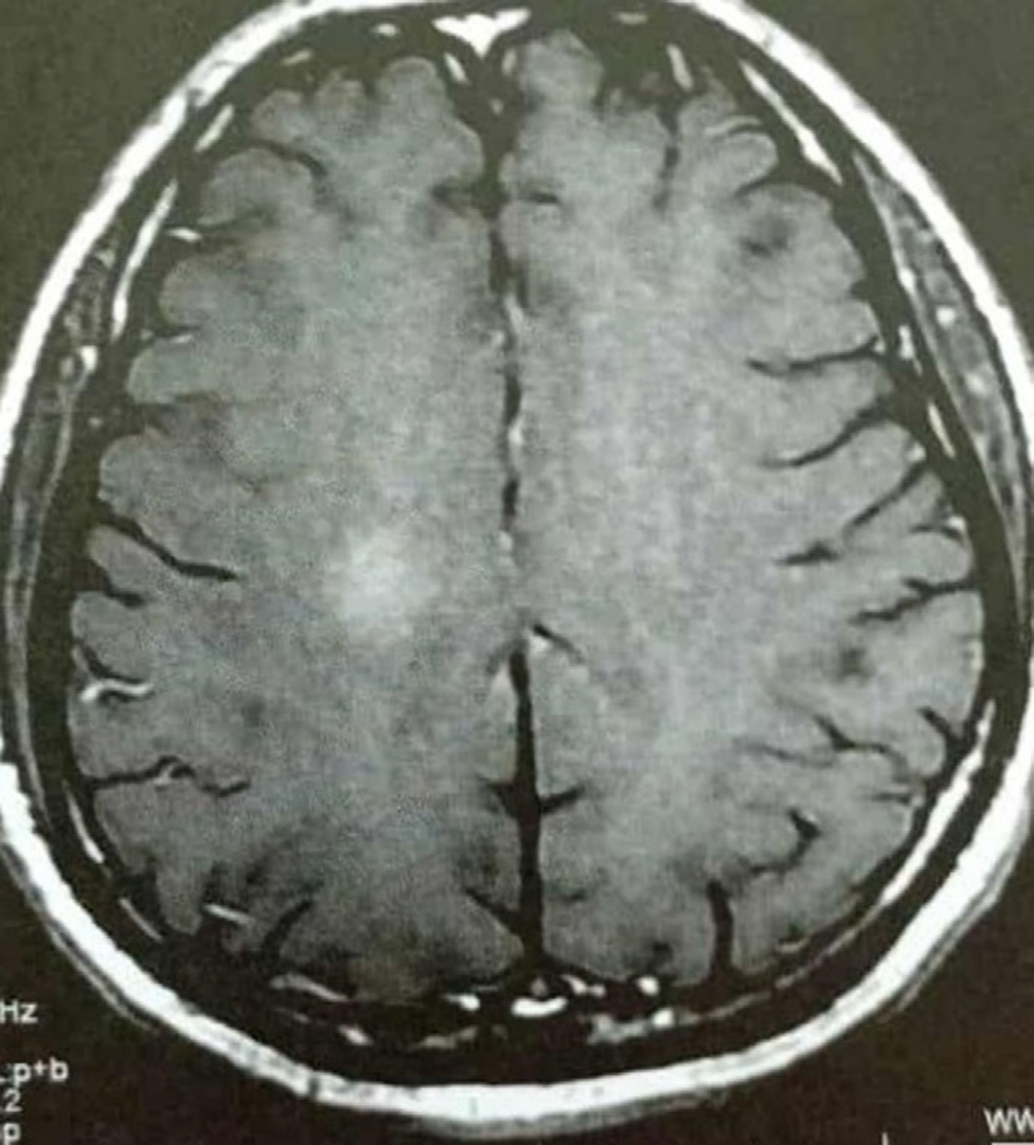

**Image 2:**
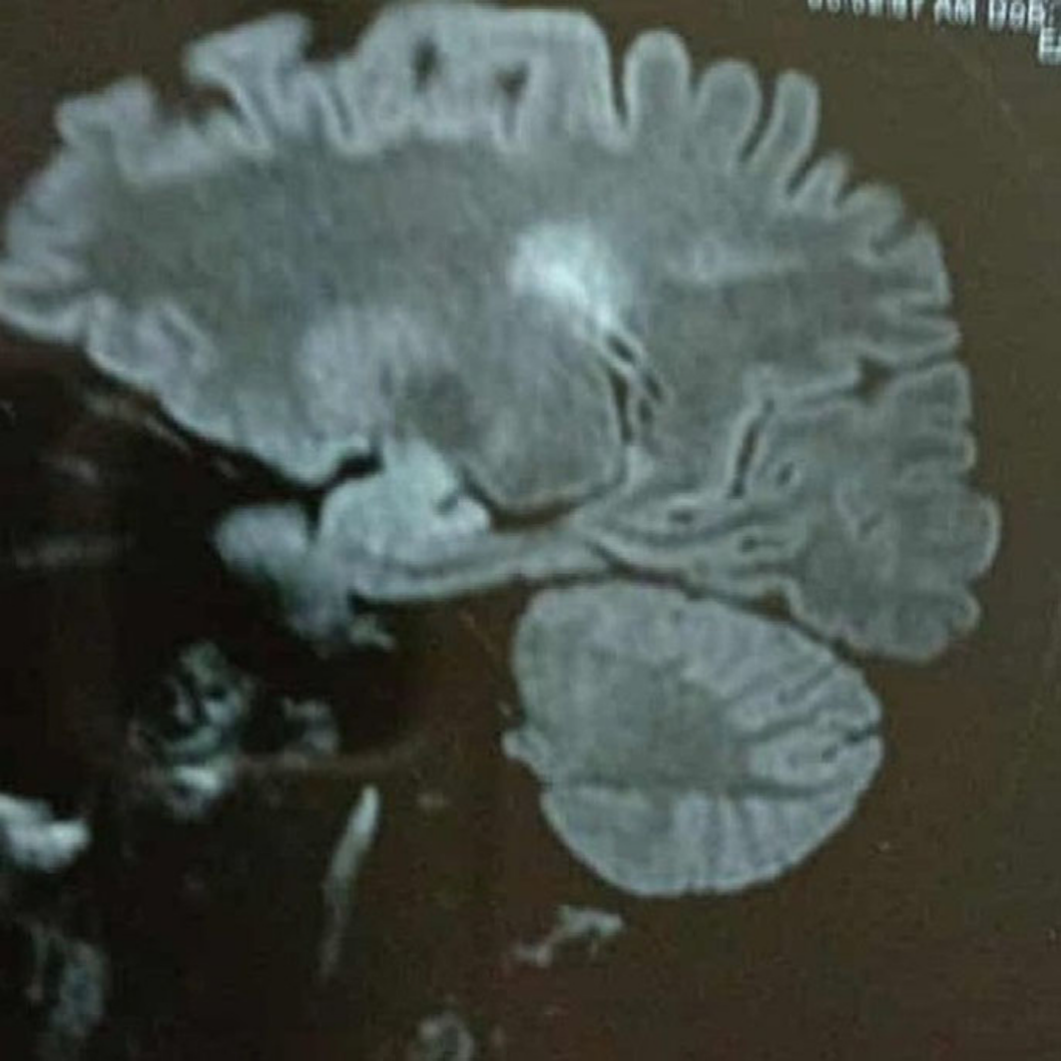

**Conclusions:**

This case highlights the importance of thorough organic work-up in late-onset psychiatric presentations.

**Disclosure of Interest:**

None Declared

